# Correction: eIF2B conformation and assembly state regulate the integrated stress response

**DOI:** 10.7554/eLife.103865

**Published:** 2024-10-03

**Authors:** Michael Schoof, Morgane Boone, Lan Wang, Rosalie Lawrence, Adam Frost, Peter Walter

**Keywords:** Human

Schoof M, Boone M, Wang L, Lawrence R, Frost A, Walter P. 2021. eIF2B conformation and assembly state regulate the integrated stress response. *eLife* **10**:e65703. doi: 10.7554/eLife.65703.

Published 10 March 2021

In the published article, there is an error in the inline Table 2 for Oligos and sgRNAs. The Sequence for sgMS006 was incorrectly shown as “GTGTGTGGTTGTCATTAGGG” which is the sequence used to target eIF2α, not eIF2Bα. The correct sequence is “GAGGACGCCATGGACGACAA” and the correct target is eIF2Bα not eIF2β as erroneously stated in the table.

The corrected Table 2 is shown here:

**Table 2. Oligos and sgRNAs.**

**Table:** 

Oligo	Sequence	Use
MS266	/5InvddT/G*G*G*A*A*CCTCTTCTGTAACTCCTTAGC	Amplify HDR template
MS267	/5InvddT/C*C*T*G*A*G*GGCAAACAAGTGAGCAGG	Amplify HDR template
MS269	TCGTGCCAGCCCCCTAATCT	Validate eIF2Bα tagging
MS270	CTGAACGGCGCTGCTGTAGC	Validate eIF2Bα tagging
MS256	AGTGAACTCTACCATCCTGA	Validate eIF2Bβ tagging
MS258	TTAGGTGGACTCCTGTGC	Validate eIF2Bβ tagging
MS096	CTGGCTAACTGGCAGAACC	Validate eIF2Bδ tagging
MS268	AGAAACAAAGGCAGCAGAGT	Validate eIF2Bδ tagging
sgMS001	CAATCTGCTTAGGACACGTG	Target Cas9 to eIF2Bβ C-terminus
sgMS004	AGAGCAGTGACCAGTGACGG	Target Cas9 to eIF2Bδ C-terminus
sgMS006	GAGGACGCCATGGACGACAA	Target Cas9 to eIF2Bα N-terminus
HDR: homology-directed recombination.		

The originally published Table 2 is shown for reference:

**Table 2. Oligos and sgRNAs.**

**Table:** 

Oligo	Sequence	Use
MS266	/5InvddT/G*G*G*A*A*CCTCTTCTGTAACTCCTTAGC	Amplify HDR template
MS267	/5InvddT/C*C*T*G*A*G*GGCAAACAAGTGAGCAGG	Amplify HDR template
MS269	TCGTGCCAGCCCCCTAATCT	Validate eIF2Bα tagging
MS270	CTGAACGGCGCTGCTGTAGC	Validate eIF2Bα tagging
MS256	AGTGAACTCTACCATCCTGA	Validate eIF2Bβ tagging
MS258	TTAGGTGGACTCCTGTGC	Validate eIF2Bβ tagging
MS096	CTGGCTAACTGGCAGAACC	Validate eIF2Bδ tagging
MS268	AGAAACAAAGGCAGCAGAGT	Validate eIF2Bδ tagging
sgMS001	CAATCTGCTTAGGACACGTG	Target Cas9 to eIF2BβC-terminus
sgMS004	AGAGCAGTGACCAGTGACGG	Target Cas9 to eIF2Bδ C-terminus
sgMS006	GTGTGTGGTTGTCATTAGGG	Target Cas9 to eIF2β N-terminus
HDR: homology-directed recombination.		

The article has been corrected accordingly.

